# Draft Genome Sequence of *Thermodesulfovibrio aggregans* TGE-P1^T^, an Obligately Anaerobic, Thermophilic, Sulfate-Reducing Bacterium in the Phylum *Nitrospirae*

**DOI:** 10.1128/genomeA.00089-16

**Published:** 2016-03-10

**Authors:** Norihisa Matsuura, Akiko Ohashi, Dieter M. Tourlousse, Yuji Sekiguchi

**Affiliations:** Biomedical Research Institute, National Institute of Advanced Industrial Science and Technology (AIST), Tsukuba, Ibaraki, Japan

## Abstract

We report a high-quality draft genome sequence of the type strain (TGE-P1^T^) of *Thermodesulfovibrio aggregans*, an obligately anaerobic, thermophilic, sulfate-reducing bacterium in the phylum *Nitrospirae*. The genome comprises 2.00 Mb in 16 contigs (3 scaffolds), has a G+C content of 34.5%, and contains 1,998 predicted protein-encoding genes.

## GENOME ANNOUNCEMENT

Currently, five species of the genus *Thermodesulfovibrio* have been validly described, namely, *T. aggregans* (1), *T. hydrogeniphilus* (2), *T. islandicus* (3), *T. thiophilus* (1), and *T. yellowstonii* (4). All five species are known to be obligately anaerobic, curved rod-shaped, thermophilic bacteria that reduce sulfate and other sulfur compounds. Physiological variability among the five species, such as substrate utilization range and the ability to perform syntrophic growth with hydrogenotrophic methanogens, has been observed. However, the genetic basis underlying these phenotypic differences remains largely unknown. In addition to other published *Thermodesulfovibrio* genomes, a high-quality genome of the type strain of *T. aggregans* will provide an important reference point to characterize the functional and genomic basis differentiating the species. With this aim, the genome sequence of the type strain of *T. aggregans* (strain TGE-P1^T^, JCM 13213^T^, DSM 17283^T^) was determined.

Genomic DNA was extracted from *T. aggregans* TGE-P1^T^ cells and sequencing libraries were prepared using the Nextera XT and Nextera mate-pair library preparation kits, with insert sizes of 200 to 700 bp and 1 to 14 kb, respectively. Sequencing was performed using an Illumina NextSeq instrument (2 × 150 bp reads) at 200× and 50× coverages for paired-end and mate-pair libraries, respectively. Raw reads were quality trimmed and filtered using Trimmomatic v0.32 (5). Trimmed reads from the paired-end library were merged with FLASH v1.2.11 (6); resulting merged and unmerged reads were used for further analyses. Quality trimmed reads from the mate-pair library were processed with NextClip v1.3.1 (7); the resulting reads in categories A, B, and C were used. The processed reads were assembled using SPAdes v3.6.0 (8). Further automated scaffolding with Opera v2.0 (9) and manual refinement of the assembly (10) using the mate-pair data were additionally performed. Gaps in the resulting scaffolds were closed using GMcloser v1.5 (11) and GapFiller v1.11 (12). Genome annotation was performed within the Integrated Microbial Genomes (IMG) platform (13).

The final high-quality, nearly complete draft assembly consists of 16 contigs in 3 scaffolds. The sequence length was 2.00 Mb, with a G+C content of 34.5%. The genome was predicted to contain 1,998 protein-coding sequences, 46 tRNA genes, and a single complete rRNA operon. Genome completeness was verified based on a lineage-specific single-copy marker gene set for the phylum *Nitrospirae* using CheckM v1.0.3 (14), resulting in detection of 669 genes out of the 676 genes (98.2% completeness). This degree of completeness is largely consistent with the range found in previously published *Thermodesulfovibrio* genomes (97.5 to 99.7% for CP001147, AUIU01000001, AXWU01000001, and IMG 2574179746), except for another *T. aggregans* JCM 13213 draft genome data (82.0%, BBCX01000001). Future comparative analysis of the *Thermoanaerovibrio* genomes will provide insight into the diverse traits of sulfate-reducing bacteria, including the genetic features responsible for the ability to perform syntrophic growth with methanogens.

### Nucleotide sequence accession numbers.

This whole-genome shotgun project has been deposited at DDBJ/EMBL/GenBank under the accession number BCNO00000000 (BioProject number PRJDB4393). The version described in this paper is version BCNO01000000.
